# Efficacy of Scapular Functional and Cervical Isometric Exercises in the Management of Chronic Mechanical Neck Pain: A Randomized Comparative Trial

**DOI:** 10.1155/prm/5873384

**Published:** 2024-12-19

**Authors:** Nasrin Bharti, Hashim Ahmed, Shahnaz Hasan, Amir Iqbal, Shadab Uddin, Waseem M. Ahamed, Fuzail Ahmad, Md. Ali Mujtaba, Ahmad H. Alghadir

**Affiliations:** ^1^Department of Physiotherapy, Buddha Paramedical College, Gida, Gorakhpur, Uttar Pradesh 273209, India; ^2^Department of Medical Rehabilitation Sciences, College of Applied Medical Sciences, Najran University, Najran 1988, Saudi Arabia; ^3^Department of Physical Therapy and Health Rehabilitation, College of Applied Medical Sciences, Majmaah University, Al-Majmaah 11952, Saudi Arabia; ^4^Department of Rehabilitation Sciences, College of Applied Medical Sciences, King Saud University, Riyadh 11433, Saudi Arabia; ^5^Department of Physical Therapy, College of Nursing and Health Sciences, Jazan University, Jazan 45142, Saudi Arabia; ^6^Respiratory Care Department, College of Applied Sciences, Al Maarefa University, Ad Diriyah 13713, Saudi Arabia; ^7^Department of Pharmaceutics, Faculty of Pharmacy, Northern Border University, Arar 91431, Saudi Arabia; ^8^Center for Health Research, Northern Border University, Arar 91431, Saudi Arabia

**Keywords:** chronic neck pain, functional disability, mechanical pain, restricted ROM, strengthening exercises, work-oriented training

## Abstract

**Background:** The global rise in work-related musculoskeletal ailments has led to issues like neck discomfort, scapular muscle dysfunction, reduced neck mobility, and functional limitations. This study aimed to evaluate the effectiveness of scapular functional exercises (SFE) and cervical isometric exercises (CIE) on pain, cervical range of motion (CROM), and functional limitations in individuals with chronic mechanical neck pain (CMNP).

**Methods:** A two-arm, parallel group pretest-post-test randomized comparative trial was conducted. Thirty participants (21 females, 9 males; average age 28.94 ± 3.77 years) were randomly divided into two groups, 1 and 2 (*n* = 15/group). Both groups received common treatments of CIE and hot packs, while Group 1 was given SFE additionally. To assess the outcomes, which included pain, cervical range of motion (CROM), and functional limitations, measurements were taken using a numeric pain rating scale (NPRS), a standard universal goniometer, and a neck disability index (NDI) questionnaire at the beginning of the study and 4 weeks after the interventions. A one-way multivariate followed by univariate analysis of covariance (MANCOVA and ANCOVA) was applied to examine the outcomes disparities within-group and between-group, with a significance level at 95% (i.e., *p* < 0.05).

**Results:** MANCOVA analysis revealed a significant impact of interventions on CROM in all directions, NPRS, and NDI scores, even after adjusting for initial scores (*F* (8, 13) = 90.1; *p*=0.001). Univariate ANCOVA showed significant improvements in outcomes for Group 1 compared to Group 2.

**Conclusions:** Adding SFE to CIE and conventional physiotherapy was more effective than just using CIE and conventional physiotherapy alone. This approach better alleviated neck pain, improved CROM (particularly in forward and left-side flexion), and reduced functional limitations in individuals with CMNP.

**Trial Registration:** ClinicalTrials.gov Identifier: NCT05624021.

## 1. Introduction

The escalating incidence of work-related musculoskeletal disorders and their consequential manifestations, such as neck pain, restricted neck movements, and functional limitations, has been well-documented in the literature [1, 2]. Studies conducted previously revealed a significant rise in the prevalence of these disorders across diverse occupational sectors, underscoring the urgent need for effective therapeutic interventions [3, 4]. Moreover, recent researches highlighted the intricate relationship between diminished muscle strength and increased susceptibility to musculoskeletal issues, further emphasizing the importance of targeted exercise-based solutions [5, 6].

In response to this burgeoning issue, deep cervical flexor (DCF) muscles were targeted with various forms of exercises with or without biofeedback devices and found to be significantly effective in managing neck pain, functional limitation, and forward head posture correction [7–10]. Investigations into the efficacy of cervical isometric exercises (CIE) as a potential remedy have gained momentum. A randomized controlled trial demonstrated that participants who engaged in a structured CIE regimen experienced a notable reduction in neck pain and enhanced cervical range of motion (CROM) compared to the group [11]. Similarly, a recently published study corroborated these findings, revealing that CIE not only alleviated discomfort but also contributed to improved muscle strength and proprioception, thereby supporting everyday activities in individuals with work-related musculoskeletal disorders [12, 13].

Additionally, current research indicates that activities targeting the scapulothoracic area and the neck may be more useful for managing individuals with NP [14]. There is limited evidence regarding the efficacies of exercises in addressing both short-term and long-term pain and functional improvements in individuals with chronic neck problems or disorders. These exercises often involve stretching and strengthening routines targeting areas like the cervical, shoulder, or thoracic regions [15–19]. However, these treatments not always alone effective for enhancing neck muscle strength [20, 21].

Therefore, the objective of this study was to evaluate the combined efficacies of scapular functional exercise (SFE) and CIE on pain levels, CROM, and functional limitations in individuals with chronic mechanical neck pain (CMNP). The hypothesis was that this combined program would be more effective than CIE alone.

By building upon this existing body of evidence, this study aims to fill a critical research gap and contribute to the understanding of an integrated therapeutic approach. Through a focused exploration of the impacts of SFE and CIE, this research endeavors to provide further insight into the possible benefits of these interventions in addressing pain, CROM, and functional limitations, thereby offering valuable guidance for clinicians and individuals seeking evidence-based approaches to manage and ameliorate the challenges associated with work-induced musculoskeletal disorders.

## 2. Materials and Methods

### 2.1. Study Design

This study employed a two arm parallel groups pretest-post-test randomized comparative design. Two groups had 30 participants (*n* = 15/group) and two measurements of the study's outcomes at baseline and 4-week postinterventions.

### 2.2. Ethical Consideration

The ethics subcommittee of King Saud University approved the study (file ID: RRC-2019-17), which was conducted in accordance with the Helsinki principles (2013) while upholding human rights. A signed informed-consent form was received from each participant prior the start of the study.

### 2.3. Study Setting

The participants with CMNP diagnosed by an orthopedic surgeon were recruited from the outpatient department physiotherapy (PT) of Integral University Hospital (Lucknow), India. The authors used posters and banners in and outside the premises of the hospital to inform the patients about the study and its implications. The study was completed within seven months, starting from August 2019 ended in March 2020.

### 2.4. Sample Size Estimation

The G∗Power 3.1.9.7- a sample size calculator, was used to calculate the sample size for this study. The mean outcome scores for the functional limitation of a sample of six participants (3/group) were utilized to obtain the intervention's effect size (Cohen's d value) in a pilot study. Using two independent sample means (N1 and N2) with standard deviation (SD), that is, *N*1 = 13.10 ± 2.21 and *N*2 = 15.27 ± 1.26, the effect size was obtained (*d* = 1.21). A *t*-test for two independent means using the power analysis type a priori: computer required sample size (2-tailed), given the power at 0.80 (80%), error probability *α* at 0.05, the allocation ratio N1/N2 = 1, and *d* = 1.21; obtained a total sample of 24 (12/group). With 20% sample attrition, 30 participants were required to satisfy the actual power of the sample.

### 2.5. Study Participants

A total of 30 individuals were randomly assigned into two groups, Groups 1 and 2 (*n* = 15/group), using the online tool “Randomization.com” (https://www.randomization.com). The inclusion criteria encompassed both males and females aged between 22 and 35 years, individuals experiencing neck pain for a duration of 3 to 12 months, absence of neck pain symptoms extending beyond the shoulder region, will to partake, and not received the clinical treatment for neck pain within the past six-month. Exclusion from the study was warranted if participants had a cervical spinal stenosis diagnosis, exhibited serious pathological conditions (e.g., inflammatory diseases, neoplasms, and fractures), suffered from upper extremity radiculopathy, had undergone before cervical spine surgery, showed evidence of nerve root compression, were pregnant, or displayed poor cooperation.

### 2.6. Procedure

**Figure 1:**
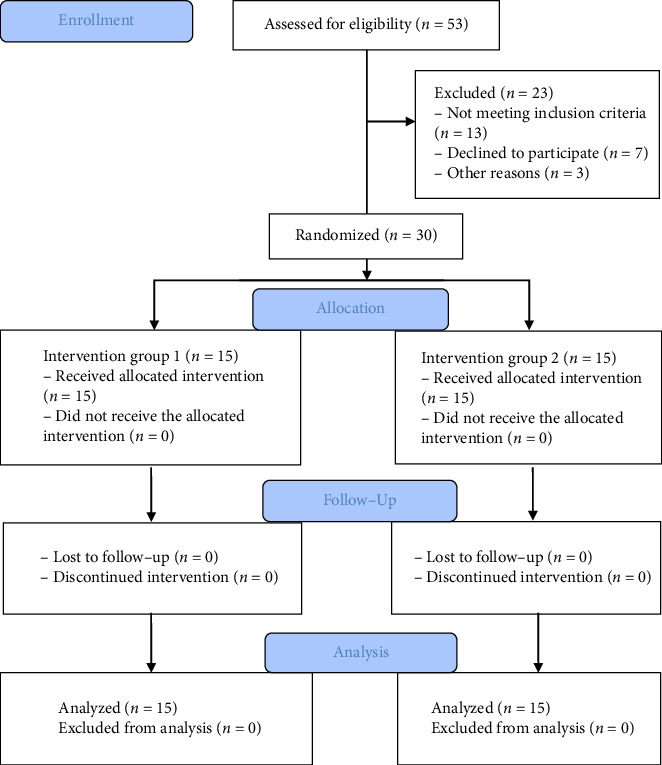
A CONSORT (2010) flow diagram presenting the study's procedures, including enrollment, assessment, randomization, allocation to intervention groups, follow-up, and study analysis.

Prior to the recruitment, the physiotherapist provided a comprehensive elucidation of the study's aims and objectives. Additionally, a formal informed consent form was administered to all participants, whereby the protocol was thoroughly explained. Patients in the Group 1 underwent SFE, CIE, and conventional PT treatment. In contrast, those in the Group 2 were given only CIE and conventional PT treatment. Prior to receiving the designated intervention based on their respective groups, the physiotherapist, who was kept blind to the study group allocation, evaluated the participants to conduct the baseline assessment for demographic parameters and the outcome measures of the study. The participants executed their designated intervention plan in accordance with their group allocation, under the observation of two senior physiotherapists who were unaware of the research group allocation. The data at 4 weeks postintervention was gathered by the same tester for a second occasion. The CONSORT (2010) flow diagram illustrates the processes involved in the research, including enrollment, allocation, follow-up, and study analysis, as presented in Figure 1.

### 2.7. Outcome Measures

The study evaluated participants' neck pain, CROM, and functional limitations using the numeric pain rating scale (NPRS), a standard goniometer, and the neck disability index (NDI) questionnaire, respectively, at the beginning and end of the 4-week period. The assessment for each outcome measure was carried out at baseline and 4 weeks postintervention.

### 2.8. Measurements

#### 2.8.1. Pain Intensity

The NPRS, a well-accepted and validated method for assessing pain intensity in clinical contexts, was used in this study. The scale consists of 11 points, ranging from 0 to 10, representing the absence of pain and the most severe pain imaginable, respectively [22]. The participants used the NPRS scale to assess and assign ratings to their present, maximum, and minimum degrees of pain experienced over the preceding 24-h period. The lowest detectable change and clinically meaningful difference for cervical pain, as measured by the NPRS, are reported to be 2.1 points and 1.3 points, respectively [23].

#### 2.8.2. Functional Status

The NDI, a self-reported questionnaire, was used to evaluate the participant's capacity to function by measuring the extent to which they have trouble moving their heads and necks [19, 20]. It is a 10 items questionnaire regarding pain during everyday activities including personal care, lifting, reading, headaches, attention, work status, driving, sleeping, and recreation [19, 20]. The intraclass correlation co-efficient (ICC) showed that the NPRS (ICC = 0.76; 95% CI, 0.51–0.87), cervical ROM (ICC = 0.86, 95% CI, 0.67–0.96), and NDI (ICC = 0.50; 95% CI, 0.25–0.67) had high test-retest reliability [22–24].

#### 2.8.3. CROM

**Figure 2:**
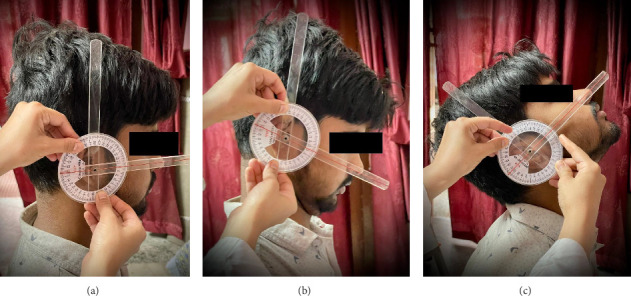
Shows the measurement of the cervical active ROM: (a) starting position of the neck in a neutral posture, (b) cervical flexion angle, and (c) cervical extension angle.

**Figure 3:**
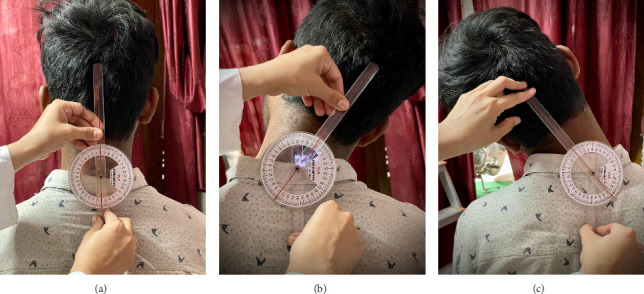
Shows the measurement of the cervical active ROM: (a) starting position of the neck in a neutral posture, (b) cervical right flexion angle, and (c) cervical left flexion angle.

**Figure 4:**
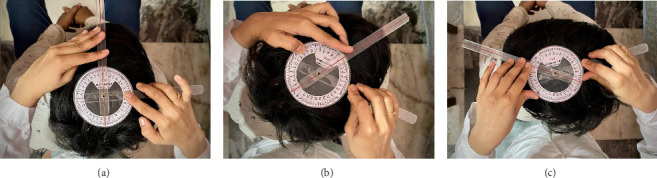
Shows the measurement of the cervical active ROM: (a) starting position of the neck in a neutral posture, (b) cervical right rotation angle (c) cervical left rotation angle.

For the assessment of cervical active range of motion, a standard full-circle goniometer (National 360 Goniometer, National Tools Ltd., located at Shop No.-1460, Daryaganj, New Delhi, India) was employed [25–27]. The measurements were conducted with the participants seated comfortably, maintaining their head in a neutral position. The patient's trunks were fastened to the chair to prevent movements of the thoracolumbar spine during the cervical movements. To measure cervical flexion and extension (as shown in Figure 2), participants were seated on a wooden chair with back support, maintaining their head in a neutral position, and hands resting on their thighs. Starting from this seated position, individuals were instructed to perform full flexion and extension of their neck, allowing us to record their CROM. During this process, the goniometer's axis was placed directly above the external auditory meatus, with the moving arm aligned with the nostrils and the fixed arm oriented vertically to the ground. For the measurement of side flexion (illustrated in Figure 3), the goniometer's axis was positioned over the spinous process of C7, the moving arm was aligned with the head's dorsal midline using the occipital protuberance as a reference; the fixed arm was placed along the thoracic spinous process and subjects were asked to flex their neck, to either side laterally. For the measurements of cervical rotation to right and left side (as shown in Figure 4), the goniometer's axis was positioned at the vertex of the head. The stationary arm was aligned parallel to a line connecting the two acromial processes, while the moving arm was directed toward the tip of the nose [25–27].

### 2.9. Interventions

#### 2.9.1. Conventional PT

Before starting the specific treatment, participants in both groups underwent a conventional PT intervention using a hydrocollator hot pack applied to the posterior neck area while lying in a supine position during their clinic visits. The hydrocollator machine was set to an optimal temperature of 78°C to ensure therapeutic heating. The hot pack was wrapped in a cotton towel to allow gradual heat release, ensuring a longer duration of therapeutic warmth. The intensity of the heat was adjusted based on the patient's tolerable warmth maintained by adding or removing towel layers. Each session lasted 25–30 min, administered once daily, five days a week, for a duration of four weeks.

#### 2.9.2. CIE

After the administration of a hot pack, participants in the Group 1 engaged in supervised CIE. The recommended exercises, designed to be executed daily over a period of 4 weeks, focus on motions specific to the neck *region*, (including neck flexion, extension, either side of lateral bending/flexion, left, and right rotation), clockwise and anticlockwise shoulder rotation, scapular retraction, and cervical retraction. Each exercise was performed based on the participant's level of discomfort and tolerance, using maximal voluntary contractions. Each contraction was held progressively for 3, 5, 7, and 10 s, with each duration repeated 10 times per session. A 30-s gap was provided between consecutive contractions. The exercises were performed once daily, 5 days a week, for a total of 4 weeks [28–30].

#### 2.9.3. SFE

The subjects in the SFE Protocol involved two key exercises: The press-up and the push-up plus [31, 32]. In the press-up, participants started in a seated position on a bench, with feet on the floor and hands on the bench's edge. From this position, they lifted their body off the bench by straightening their arms, and then dipped down near the bench seat, moving mainly their shoulder girdle. To advance the difficulty of this exercise, weight plates were added on the thighs [31, 32].

The push-up plus was executed starting from a standard push-up position, either on hands and feet or knees. Participants maintained a rigid torso by bracing their abdominals. The key movement was to push the body as high as possible off the floor by protracting the scapula. This exercise was intensified first by switching from knees to feet and then by adding iron plates on the upper back for added resistance [31, 32].

Both press-up and push-up plus exercises, as part of the SFE program, were performed 10 times per session per day without any gap between consecutive repetitions. The exercises were conducted 5 days a week for a duration of 4 weeks.

The study evaluated participants' pain intensity, CROM, and neck functional status using the NPRS, a standard goniometer, and the NDI questionnaire, respectively, at the beginning and end of the 4-week period [19, 20, 22, 23, 25–27].

### 2.10. Statistical Analysis

A statistical tool, computer software IBM SPSS v.23, was used to analyze the data. The noncontinuous variables, such as the frequency and percentage of the demographic factors, such as gender, were examined using a Chi-square test. A Levene's test for equality of error variances is conducted before performing a MANOVA (Multivariate Analysis of Variance) to ensure that the assumption of homogeneity of variances is met, which is crucial for the validity of MANOVA results. A one-way MANCOVA test was used for between-subject effect within each group and follow-up univariate ANCOVAs was conducted pairwise comparison between the treatment groups for post-test scores of all the dependent variables (mean difference). The significance level *p* value was set at less than 0.05 for the differences to be considered significant.

## 3. Results

**Figure 5:**
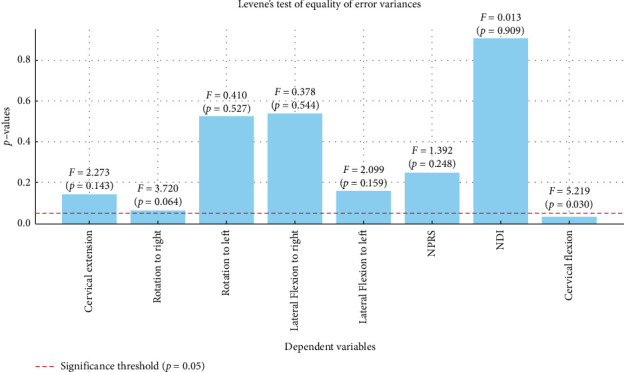
Visually illustrates the results of Levene's test for equality of error variances across various dependent variables. The *p* values for each variable are displayed, with a red dashed line indicating the significance threshold of *p*=0.05.

Data for the participants' age and continuous variables were normally distributed in both groups. Participants from both Groups 1 and 2 were matched for the age and the outcomes scores of cervical ROM, NPRS, and NDI at baseline, using an unpaired *t*-test (*p*=0.79). Similarly, a Chi-square test revealed a matched distribution for female and male participants between the groups (*p*=0.69). The demographic characteristics and baseline values are presented in Table 1. Furthermore, the results of Levene's test for equality of error variances across multiple dependent variables are presented in Figure 5.

Table 2, illustrating a statistically significant multivariate effect of the group on the combined dependent variables after controlling for pretest scores, *F* (8, 13) = 90.053, *p* < 0.001, Wilks' Λ = 0.018, indicating a large effect size. The group explained a substantial amount of variance in the combined dependent variables, with a very strong association.

**Figure 6:**
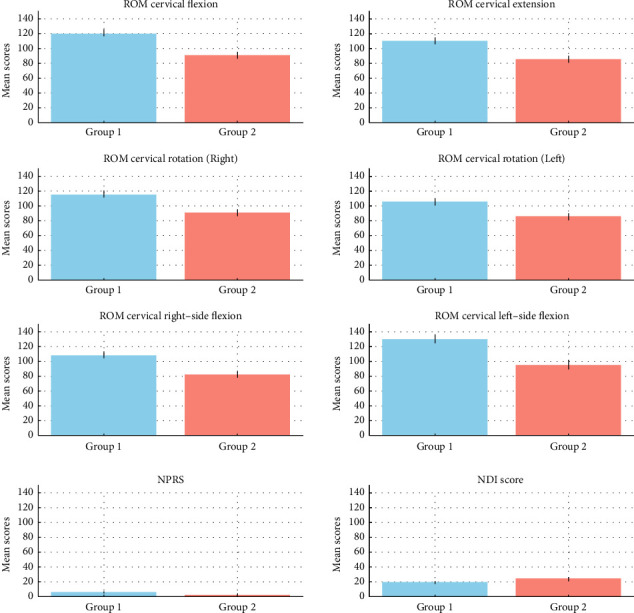
Visually illustrate the significant differences in outcomes between the two groups across various measures, highlighting the large effect sizes in most of the analyzed parameters.

Follow-up univariate ANCOVAs revealed significant differences between Group 1 (SFE with CIE) and Group 2 (CIE) across several post-test outcomes (Figure 6). Group 1 had significantly higher adjusted means for post-test ROM cervical flexion (*F* (1, 20) = 123.315, *p* < 0.001), cervical extension (*F* (1, 20) = 30.923, *p* < 0.001), cervical rotation to the right (*F* (1, 20) = 48.192, *p* < 0.001), cervical rotation to the left (*F* (1, 20) = 15.401, *p*=0.001), cervical right-side flexion (*F* (1, 20) = 85.923, *p* < 0.001), and cervical left-side flexion (*F* (1, 20) = 194.105, *p* < 0.001), all indicating large effect sizes. The NPRS also showed significant differences (*F* (1, 20) = 47.059, *p* < 0.001) with a large effect size. Although the NDI score differed significantly between groups (*F* (1, 20) = 4.981, *p*=0.037), the effect size was smaller compared to other measures.

A Bonferroni adjustment for multiple comparisons was applied, with statistical significance accepted at *p* < 0.00625 (0.05/8 dependent variables). All reported *p* values were below this threshold, confirming the robustness of the findings (Table 3).

## 4. Discussion

This study compared the effectiveness of SFE versus CIE and conventional PT in treating patients with CMNP. A total of 60 patients participated, divided evenly between two groups. Group 1 received SFE alongside CIE and conventional PT, while Group 2 received only CIE and conventional PT. The outcomes were assessed using a multidimensional approach. Pain intensity and functional limitations were evaluated through the NPRS and the NDI questionnaire, respectively. CROM was measured using a standard full-circle goniometer, ensuring precise and consistent data collection. This comprehensive evaluation provided insights into the comparative effectiveness of the two treatment approaches in addressing pain, function, and mobility in patients with CMNP.

The findings of the present study indicate that patients in Group 1 with CMNP, who received SFE in addition to CIE and conventional PT, showed significant improvements in pain intensity, functional limitations, and CROM as assessed by the NPRS, NDI, and goniometer measurements. While both groups experienced reductions in pain intensity, Group 1 demonstrated a greater magnitude of improvement. At baseline, the two groups had comparable measurements, ensuring that the observed changes in pain intensity and other outcomes could be attributed directly to the interventions. SFE, such as press-up and push-up plus are known to effectively engage the lower trapezius and serratus anterior muscles, which play a critical role in stabilizing and positioning the scapula [33]. When the serratus anterior does not function properly, scapular stability is compromised, leading to poor alignment [34]. This misalignment can place abnormal pressures on the cervical and thoracic spine, potentially worsening or prolonging neck pain [35].

The NPRS scores in this study demonstrated a significant reduction, surpassing the Minimal Detectable Change (MDC) of 2.1 points reported in prior research, highlighting the clinical significance of the improvement [36]. Moreover, the within-group average change scores for both Groups 1 and 2 exceeded the Minimum Clinically Important Difference (MCID), further emphasizing the relevance of these findings. It is supported by a recently published systematic review and meta-analysis which concluded that scapular-targeted therapy significantly alleviated pain intensity in patients with chronic neck pain, particularly among women [37].

Similarly, earlier studies on the NDI have confirmed its acceptable responsiveness and fair to moderate test-retest reliability. These studies also suggested that the MCID for the NDI could be nearly double the previously established value of 19 points, reinforcing the significance of the observed outcomes and the effectiveness of SFE as part of a comprehensive treatment strategy for CMNP [36]. In this study, neck disability decreased significantly in both groups, although the magnitude of decrement was higher in the Group 1. The MDC of NDI is reported to be 5 points out of 50 based on 95% of CI [38]. In our study, this change is far beyond five points. MacDermid et al., 2009 described MCID for NDI as 14%. In the Group 1, the decrease was 66.66%, which depicts much relief in patients with MNP compared to 43.05% in the Group 2. Changes in neck disability on the self-reported NDI scale were remarkably better in the Group 1 [38]. In contrast, Chen et al. observed that although scapular-targeted therapy effectively reduced subjective pain intensity, it failed to yield significant improvements in neck disability scores or pain pressure thresholds. This discrepancy suggests that alleviating perceived pain does not always correlate with enhanced functional outcomes or measurable changes in pain sensitivity [37].

In this current study, both groups experienced a noticeable increase in the CROM. Previously published studies advocated that the patients with neck pain experienced a noticeable improvement in their ROM [39–41]. In contrast, a recently published study reported on patients with neck pain, found no connection between ROM and clinical relief [42]. Similarly, another study highlighted that the impact of scapular targeted therapy on cervical ROM remains uncertain due to limited supporting evidence, suggesting a need for further research to establish definitive conclusions [37].

Additionally, studies show that consistent hot packs application over 4 weeks can effectively complement other therapeutic exercises and interventions, thus adds in improving pain and relaxing the muscles by removing chemical mediators and increasing blood flow to the tissues [43, 44].

The findings corroborated earlier research that showed pain might be lessened by strength and endurance training. Additionally, it has been demonstrated that general workouts like strength and endurance training and particular exercises like craniocervical flexion of DCF might lessen neck pain [29, 32]. In this study, the strengthening and endurance training components of the SFE likely played a key role in giving Group 1 an advantage over Group 2. This advantage was observed in terms of reduced pain, improved functional limitations, and enhanced CROM in Group 1.

### 4.1. Study Limitations

The study was limited in terms of the potential for bias in the outcomes due to the therapist's lack of blinding to group assignment; lack of follow-up period to ensure the long-term effect of the intervention, and all samples was taken from a relatively restricted population that might affect the generalization of the study's report to worldwide. Therefore, future research should have the specialist physiotherapists per the number of groups in the study to avoid the chances of potential bias, a follow-up period to observe whether these clinical benefits persist over a more extended period and include the participants from a more extensive mixed population.

## 5. Conclusion

The results of this study indicated that individuals with CMNP who underwent SFE in addition to CIE and conventional PT showed greater improvements in reducing pain, enhancing CROM, and decreasing functional limitations compared to those who only performed CIE and conventional PT. The study suggests that focusing on the scapulothoracic region in exercise interventions can yield beneficial outcomes for patients with this condition. Furthermore, the insights gained from this research provide healthcare professionals with enhanced understanding, emphasizing the importance of integrating SFE in addition to CIE and conventional PT in the treatment of CMNP.

## Figures and Tables

**Table 1:** Shows the matching of demographic characteristics of the participants and baseline outcome scores, using an independent *t*-test and Chi-square test for continuous and categorical variables (*N* = 30).

Baseline scores of variables		Group 1 (*n* = 15) Mean ± SD	Group 2 (*n* = 15) Mean ± SD
Age		29.60 ± 3.39	28.27 ± 4.14

Gender	Females (%)	10 (66.67)	11 (73.33)
	Males (%)	5 (33.33)	4 (26.67)

Cervical ROM	Flexion	73.50 ± 2.69	72.87 ± 2.58
	Extension	61.87 ± 2.13	61.47 ± 2.16
	Rotation to right	77.60 ± 3.61	78.00 ± 3.58
	Rotation to left	75.73 ± 3.84	76.00 ± 3.79
	Lateral flexion to right	33.40 ± 2.44	37.73 ± 1.98
	Lateral flexion to left	34.40 ± 2.29	33.40 ± 2.44

NPRS scores		5.47 ± 0.99	5.13 ± 0.91
NDI scores		23.87 ± 1.88	24.33 ± 2.02

*Note:* N/n = total number of participants/in each group; Group 1: received SFE and CIEs; Group 2: received CIE alone.

Abbreviations: NDI, neck disability index; NPRS, numeric pain rating scale; ROM, range of motion; SD, standard deviation.

**Table 2:** Tests of between-subjects effects within the participant's treatment group, using a MANCOVA test (*n* = 15/group).

Post-test scores for dependent variables		Type III sum of squares	df	Mean square	*F* value	*p* value
Cervical ROM (°)	Flexion	148.50	1	148.50	123.315	0.001⁣^∗^
	Extension	20.87	1	20.87	30.923	0.001⁣^∗^
	Right rotation	62.95	1	62.95	48.192	0.001⁣^∗^
	Left rotation	66.84	1	66.84	15.401	0.001⁣^∗^
	Lateral flexion to right	116.67	1	116.67	85.923	0.001⁣^∗^
	Lateral flexion to left	161.74	1	161.74	194.105	0.001⁣^∗^

NPRS scores		17.18	1	17.18	47.059	0.001⁣^∗^
NDI scores		17.51	1	17.51	4.981	0.037⁣^∗^

*Note: n* = total number of participants in each group.

Abbreviations: CI, confidence interval; NDI, neck disability index; NPRS, numeric pain rating scale; ROM, range of motion; SD, standard deviation.

⁣^∗^Significant value, if *p* < 0.05.

**Table 3:** Pairwise comparison between the treatment group for post-test scores of all the dependent variables, using a univariate ANCOVA test.

Dependent variables		Mean differences	Std. error	*p* value	95% CI for difference	
					Lower bound	Upper bound
Cervical ROM	Flexion	6.994	0.630	0.001⁣^∗^	5.680	8.308
	Extension	2.623	0.472	0.001⁣^∗^	1.639	3.607
	Rotation to right	4.554	0.656	0.001⁣^∗^	3.186	5.922
	Rotation to left	4.693	1.196	0.001⁣^∗^	2.198	7.187
	Lateral flexion to right	6.198	0.669	0.001⁣^∗^	4.803	7.593
	Lateral flexion to left	7.300	0.524	0.001⁣^∗^	6.207	8.392

NPRS scores		−2.379	0.347	0.001⁣^∗^	−3.103	−1.656
NDI scores		−2.401	1.076	0.037⁣^∗^	−4.646	−0.157

*Note: n* = total number of participants in each group.

Abbreviations: CI, confidence interval; NDI, neck disability index; NPRS, numeric pain rating scale; ROM, range of motion; SD, standard deviation.

⁣^∗^Significant value, if *p* < 0.05.

## Data Availability

The datasets used and/or analyzed during the current study are available from the corresponding author upon reasonable request.
